# What Employer Actions Are Considered Most Important for the Return to Work of Employees with Cancer? A Delphi Study Among Employees and Employers

**DOI:** 10.1007/s10926-018-9800-z

**Published:** 2018-07-19

**Authors:** M. A. Greidanus, S. J. Tamminga, A. E. de Rijk, M. H. W. Frings-Dresen, A. G. E. M. de Boer

**Affiliations:** 1 Amsterdam UMC, University of Amsterdam, Coronel Institute of Occupational Health, Amsterdam Public Health research institute, Meibergdreef 9, Amsterdam, The Netherlands; 2 Department of Social Medicine, Faculty of Health, Medicine and Life Sciences, Research Institute Primary Care and Public Health (CAPHRI), Maastricht University, Universiteitssingel 40, Maastricht, The Netherlands

**Keywords:** Neoplasms, Return to work, Delphi technique, Employment, Sick leave

## Abstract

*Purpose* Employers are important stakeholders in the return to work (RTW) of employees with cancer. However, it is unclear what employer actions are most important to that process. The objective, therefore, was to reach consensus on what employer actions are considered most important for the RTW of employees with cancer, by employers and employees separately. *Methods* A two-round online Delphi study was conducted with two expert panels: one with 23 employers and one with 29 employees with cancer. The results from each panel were analysed separately. Out of 24 suggested employer actions, participants selected the 10 they considered most important for RTW in each of the following RTW phases: (1) *disclosure*, (2) *treatment*, (3) *RTW plan,* and (4) *actual RTW*. The consensus threshold was set at ≥ 80% during the second round. *Results* The employer and employee expert panels both reached consensus on the importance of ‘emotional support’, ‘practical support’, ‘allow sufficient sick leave’, ‘plan return to work’, ‘adjust expectations’, ‘assess work ability’, and ‘show appreciation’. Employers also reached consensus on ‘communicate’ and ‘treat normally’, and employees on ‘handle unpredictability’. All these employer actions were considered to be specific for one to three RTW phases. *Conclusions* Employers reached consensus on the importance of nine employer actions, employees on eight. Both stakeholder perspectives showed great similarities, but did vary regarding important employer actions during the employee’s treatment. We recommend developing interventions targeting the employer, meeting both employer and employee needs in each RTW phase, to enhance RTW support for employees with cancer.

## Background

The current worldwide incidence of cancer is 14.1 million and this number is expected to increase to an annual 25 million new diagnoses in 2025 [1, 2]. Approximately 50% of those diagnosed with cancer are of working age [3]. This percentage also likely to increase considerably, mainly due to the trend of employees having to work longer before reaching retirement age, while the incidence of cancer is the highest in the 65–69 age group [4]. The increased incidence rates in the working population in combination with improved survival rates imply that employees who return to work (RTW) or continue working after a cancer diagnosis are becoming more common in workplaces [5]. For these employees with cancer, participating in work is an important step forward towards a ‘normal life’, since work provides structure, the feeling of social belonging and financial security [6, 7]. However, various problems related to the employee’s workplace and mental, physical and (psycho)social functioning may impede the work participation of employees with cancer, such as fatigue, depression, problems with physical tasks and a lack of support from the workplace [6, 8–11]. On average, only 62% of employees with cancer have returned to work or are still working 1 year after diagnosis [12]. Facilitating work participation of employees with cancer is therefore an increasingly relevant topic.

Although several stakeholders can facilitate work participation of employees with cancer, attention has lately been drawn to the role of the employer during the RTW process [13–16]. The employer is designated as one of the main stakeholders since a supportive employer has been found to be a key facilitator of work participation by employees with cancer [17]. However, a recent review showed that both employees with cancer and employers perceive a plurality of barriers for work participation of employees with cancer related to the employer, e.g. employer–employee communication, employer’s knowledge about cancer and employer’s perception of the employee’s ability to work [18]. The variety of these perceived employer-related barriers indicates that being a supportive employer of employees with cancer is not straightforward. Rather, the employer has a complex and demanding role to play during the RTW process, may face different ways of influencing RTW and indicate a need for support [18, 19].

Current evidence on the role of the employer during the RTW of employees with cancer is mainly obtained by qualitative studies, and perspectives are wide and sometimes even contradictory [17, 18]. However, knowledge on what employer actions are considered most important to facilitate the RTW of employees with cancer is scarce and only descriptive in nature [20]. Ordering such actions by their perceived importance is of great relevance—among other things, in order to gain an insight into which employer actions should be prioritised in future RTW interventions targeting employers. Moreover, based on a recent qualitative study on the role of the employer during RTW of an employee with cancer [19], the RTW trajectory of employees with cancer can generally be divided into four phases: (1) *disclosure*, (2) *treatment*, (3) *RTW planning,* and (4) *actual RTW*. If we can determine what employer actions are considered most important during each of these phases, we may be able to intervene on the employers’ RTW support for employees with cancer, and thereby contribute to a sustainable work participation of employees with cancer.

The objective of this study was therefore to reach consensus on what employer actions are considered most important for the RTW of employees with cancer. The viewpoints of both employees with cancer and employers are of interest, since any differences between the two perspectives would have important implications for practice. The current study therefore mapped each perspective separately, resulting in the following research questions: what employer actions are considered most important for the RTW of employees with cancer, according to (a) employees with cancer and (b) employers?

## Methods

### Design

**Fig. 1:**
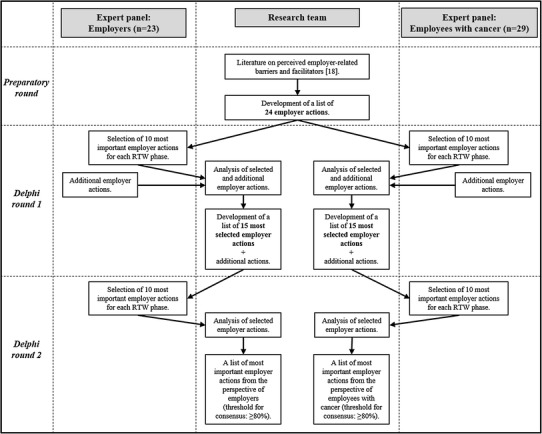
Design of the Delphi study, consisting of a *preparatory round* and two *Delphi rounds, 1* and *2*

The Delphi technique was used, with a *preparatory round* and then two *Delphi rounds, 1* and *2*. The *preparatory round* was performed by the research team on the basis of a recent systematic review [18], whilst both Delphi rounds were each performed by two expert panels (see Fig. 1). This design was defined a priori and contained four important characteristics of the Delphi technique: experts participated in the Delphi rounds ‘anonymously’; the design contained ‘iterations’ of Delphi rounds (i.e. Delphi round 1 and 2); experts were provided with ‘controlled feedback’ of group responses; and ‘statistical group responses’ were analysed [21]. As such, the design was used to obtain consensus on which employer actions are considered as most important for the RTW of employees with cancer, by two expert panels separately. The Qualtrics online questionnaire system (http://www.qualtrics.com) was used and data was analysed using SPSS software (IBM SPSS Statistics version 24).

### Expert Panels

#### Employers

The inclusion criteria for the employer expert panel were that employers had been responsible for guiding at least one employee with cancer in the past 5 years (e.g., as HR manager or supervisor) and were able to speak and read Dutch. Employers were recruited via employers’ organisations, databases of employers who have participated in previous research [19, 22], social media and snowballing techniques. We strove for a heterogeneous panel in respect of gender, company size and function (supervisors, HR personnel and other employer representatives).

#### Employees with Cancer

For the employee expert panel, the inclusion criteria were that employees had been diagnosed with cancer in the past 7 years, were over 18 years of age at time of diagnosis, worked for an employer (either part-time or full-time, on a flexible, temporary or permanent basis) at time of diagnosis and were able to speak and read Dutch. A heterogeneous (gender and diagnosis) group of employees with cancer was purposefully sampled using a database of previous research [23]. In addition, we strove for heterogeneity in respect of company size and RTW outcomes.

We aimed to have 16 experts on each expert panel during the second Delphi round, as recommended for Delphi studies with a similar aim [24, 25]. Taking into account an expected 20% loss of follow-up after the first round, we included experts until 20 experts per expert panel filled out the first Delphi round, with no cut-off point in inclusion time. Experts were recruited in April–May 2017. Before inclusion, all experts received information about the study from the first author, both online and by telephone, and signed an online informed consent form.

### Procedure

#### Preparatory Round

During this round, a list of employer actions was compiled based on a recent systematic review [18]. Consisting of 47 qualitative studies conducted from the perspective of employees with cancer and five from the employer perspective, this review identified perceived employer-related barriers and facilitators for work participation of employees with cancer. It applied the following definitions of a ‘barrier’ and a ‘facilitator’: *behaviour, attitude and perception of the employer that was perceived to hinder (barrier) or enhance (facilitator) sustainable work participation of cancer survivors* (p. 726). The first author (MG) openly coded the barriers and facilitators, resulting in several broad categories (e.g. ‘communication’, ‘practical support’ and ‘colleagues’). Subsequently, one concrete employer action per category was formulated, including a brief explanation which reflected the essence of all the barriers and facilitators in that specific category (e.g. ‘communicate: communicate effectively with the employee with cancer, in terms of tone, intensity, subjects and channels’). Next, the second author (ST) checked these first two steps. Finally, all the authors checked the complete list of employer actions, including the accompanying explanations, in respect of formulation (clear and consistent) and whether each action was indeed a concrete employer action. This process resulted in a list of 24 employer actions, which was used as input for Delphi round 1.

#### Delphi Round 1

In this first Delphi round, the experts’ demographic and work-related characteristics were recorded. Then the four RTW phases were addressed in turn.

##### **Phase 1, Disclosure**

The period between disclosure of the employee’s illness to the employer and the first treatment.

##### **Phase 2, Treatment**

The period during which the employee is on sick leave as a result of their treatment.

##### **Phase 3, RTW Planning**

The period in which concrete planning of and preparation for the employee’s RTW take place. The employee is still on sick leave during this phase.

##### **Phase 4, Actual RTW**

The period after RTW, up until 6 months after a stable work situation is reached. With ‘stable’ we refer to unchanged working hours and position at work.

These RTW phases were based on a recent qualitative study concerning the role of the employer in case an employee is diagnosed with cancer and adapted by more strict definition of the phases in consultation with all authors [19]. The period of 6 months for phase 4 was meant to make the experts aware that phase 4 was not only the moment of re-entering work, but also included a certain period of follow-up. Each phase was introduced in brief, after which the experts were asked whether they could recognise themselves in that phase. If so, the 24 employer actions were displayed in random order and the experts were asked to select the 10 they considered ‘most important for successful guidance focused on RTW’. If not, this expert was not asked to select the most important employer actions for this RTW phase and was thereby not taken along in the analysis of this RTW phase. This was done for each RTW phase separately. After each phase presentation, the experts who selected important employer actions were given the opportunity to add additional employer actions. The questionnaire of this Delphi round was pilot tested in respect of its formulation and use by four persons with a diverse level of education (low to high level of education), two of them were employees with cancer. Feedback was gathered by a telephone or face-to-face interview. Based on these pilot tests, some sentences and terminology were simplified and the experts were given the opportunity to add comments at the end of the Delphi round. The experts were asked to fill out the questionnaire of Delphi round 1 within a week, and if necessary received an e-mail and telephone reminder after 1 and 2 weeks respectively. Data collection of Delphi round 1 took place in April–May 2017.

After all experts participated in the first Delphi round, the percentage selecting a certain employer action as ‘most important’ was calculated separately for each expert panel and each RTW phase. This percentage was calculated to determine the ‘controlled feedback’ for the second Delphi round and to identify the employer actions which were selected as ‘most important’ by the highest percentage of experts. Since the purpose of Delphi round 1 was not to reach consensus among the experts, no criteria for consensus were defined for this round. In addition, four authors (MG, ST, MFD and AdB) decided by consensus whether or not to include the additional suggested actions in Delphi round 2. This decision was based on the following criteria: an included additional action indeed had to be an employer action and had to be one not already covered by any of the other actions.

#### Delphi Round 2

The 15 employer actions selected as ‘most important’ by the highest percentage of experts during the first Delphi round were included in the second Delphi round, together with additional actions that met the above criteria. The number of 15 actions was determined by the research team a priori, with the intention to incorporate a workable number of actions in the second Delphi round, without losing a sizeable range of options. In the second Delphi round, the employer actions were displayed in descending order and the percentage of experts who had selected each of them during the first round was shown (‘controlled feedback’). The questionnaire of Delphi round 2 was pilot tested by the same persons who pilot tested the questionnaire of Delphi round 1, but that exercise produced no major changes. Data was collected in May–June 2017. In this period, the experts were once again asked to select the 10 employer actions they considered ‘most important for successful guidance focused on RTW’. During the second Delphi round, experts could not add additional employer actions. All other methods for data collection were similar to the methods used for Delphi round 1.

The same procedure for data analysis was used as after the first Delphi round, with separate calculations for each RTW phase and each expert panel. Consensus was reached when ≥ 80% of the expert panel selected a certain employer action as ‘most important’ during a specific RTW phase. The research team decided a priori that two Delphi rounds were enough for the experts to make a considerate selection of the employer actions. The rationale behind this decision was that experts were given the opportunity to add additional employer actions (during Delphi round 1), were provided with controlled feedback of group responses (during Delphi round 2) and were given the opportunity to select any additional employer actions that were suggested by other experts (during Delphi round 2). Moreover, a small number of additional added employer actions during Delphi round 1 were expected, since the list of employer actions that was used as input for this round was expected to be fairly complete due to its comprehensive basis.

## Results

### Expert Panels

#### Employers

Twenty-three employers participated in the study (see Table 1), with 22 taking part in all rounds. Employers were recruited via employer’s organisations (n = 9), previous research (n = 5) [19, 22], social media (n = 1) or snowballing (n = 6). One did not answer the question about how he was recruited for the study. The employers had an average of 11 years of experience in guiding employees with cancer and collectively had worked with about 150 employees with cancer. Heterogeneity was reached in terms of gender and function, but most of the experts worked at medium-sized or large companies (≥ 51 employees). During the second Delphi round, all the employers indicated that they recognised themselves in all the RTW phases, with the exception of one and two who did not recognise themselves in phases 3 and 4, respectively, because the employees they had guided were ultimately unable to plan or achieve their RTW.

**Table 1:** Characteristics (i.e. demographics and work characteristics) of the employers on the expert panel

Employer characteristics (n = 23)	
Demographics	
Gender: male N (%)	11 (48%)
Work characteristics N (%)	
Position	
HR manager	9 (39%)
Supervisor	6 (26%)
HR advisor	5 (22%)
Other	5 (22%)
Company size	
≤ 50 Employees	2 (9%)
51–250 Employees	9 (39%)
≥ 251 Employees	12 (52%)
Experience in years: mean ± SD (range)	11 ± 7 (0–30)
Experience: number of employees with cancer	
≤ 3 Employees	10 (43%)
4–6 Employees	9 (39%)
≥ 7 Employees	4 (17%)

#### Employees with Cancer

A heterogeneous group of 29 employees completed both Delphi rounds (100% response rate). Most had been diagnosed with breast cancer (n = 9), gastro intestinal cancer (n = 6) or bladder cancer (n = 5) (see Table 2). The employees scored the guidance they had received from their employer with 3.8 ± 1.4 (1–5) on a scale of 1 (‘completely dissatisfied’) to 5 (‘completely satisfied’). During the second Delphi round, 90% recognised themselves in RTW phase 1 (*disclosure*), 90% in phase 2 (*treatment*), 86% in phase 3 (*RTW planning*) and 90% in phase 4 (*actual RTW*). The reasons cited for not identifying with a certain phase were that the employee was ‘diagnosed very suddenly and treated the same day’ (phase 1), ‘worked throughout the treatment’ (phase 2 and 3), and ‘has still not returned to work’ (phase 4). The number of employees that were included for analysis per RTW phase can be found in Table 3.

**Table 2:** Characteristics (i.e. demographics, work characteristics, educational level and diagnosis) of the employees with cancer on the expert panel

Employee characteristics (n = 29)	
Demographics	
Age: mean ± SD (range)	56 ± 9 (26–67) years
Gender: male N (%)	12 (41%)
Work characteristics N (%)	
Management position	9 (31%)
Company size	
≤ 50 Employees	8 (28%)
51–250 Employees	2 (7%)
≥ 251 Employees	19 (66%)
Current work status	
Same company, same position	17 (59%)
Same company, different position	7 (24%)
Other company	2 (7%)
Partly work disabled	2 (7%)
Completely work disabled	1 (3%)
Unemployed	1 (3%)
Retired (including early retirement)	1 (3%)
Duration of absence or partly absence due to illness: months ± SD (range)	11 ± 8 (0–36)
Educational level N (%)	
High	4 (14%)
Intermediate	14 (48%)
Low	11 (38%)
Diagnosis N (%)	
Primary diagnosis	
Breast cancer	10 (35%)
Gastro intestinal cancer	6 (21%)
Bladder cancer	5 (17%)
Other (e.g. kidney cancer, prostate cancer or leukaemia)	8 (28%)
Time since primary diagnosis: years ± SD (range)	6 ± 4 (1–16)

**Table 3:** Selection of employer actions during Delphi round 2

Selection of employer actions	Expert panel	Employers				Employees with cancer			
	RTW phase	1. Disclosure	2. Treatment	3. RTW planning	4. Actual RTW	1. Disclosure	2. Treatment	3. RTW planning	4. Actual RTW
	N	22	22	21	20	26	26	25	26
Employer actions	Brief explanation	Percentage of the expert panel selecting this action during Delphi round 2 as one of the 10 ‘most important’ employer actions							
**Support practically**	Provide the employee with cancer with practical support (e.g. adapting tasks, workplace and working hours)	**100**	73	76	**90**	**85**	69	**96**	**92**
**Assess work ability**	Assess the extent to which the employee with cancer is able to work in the right manner	73	59	**95**	**85**	**81**	69	**96**	**92**
**Show appreciation**	Give the employee with cancer the feeling that you want them back at work	73	77	**86**	**85**	65	73	**80**	**92**
**Communicate**	Communicate effectively with the employee with cancer (in terms of tone, intensity, subjects and channels)	**91**	**100**	**95**	55	73	77	72	62
**Support emotionally**	Support the employee with cancer emotionally (e.g. showing interest, being involved and understanding)	**86**	**100**	71	60	**81**	77	52	62
**Adjust expectations**	Adjust expectations regarding the performance of the employee with cancer to their current situation	41	55	**95**	70	69	73	**84**	77
**Allow sufficient sick leave**	Allow sufficient sick leave and not putting pressure on the employee with cancer to return to work	77	**100**	52	N/A	**96**	**92**	76	54
**Treat normally**	Treat the employee with cancer as if they are not ill (e.g. avoid inappropriate treatment, including being too protective or concerned)	64	50	76	**90**	69	58	60	69
**Plan return to work**	Make a plan for the employee’s RTW in consultation with them	N/A	**82**	**95**	**80**	23	38	**92**	69
**Handle unpredictability**	Try to cope as well as possible with the unpredictability of the illness and the absence of the employee with cancer	N/A	73	48	40	73	**81**	68	65
**Reduce work pressure**	Reduce the pressure of work on the employee with cancer	41	N/A	19	40	69	62	76	77
**Radiate a positive attitude**	Radiate a positive attitude when guiding the employee with cancer	45	59	57	15	58	42	32	62
**Respect privacy**	Respect the privacy of the employee with cancer	50	41	N/A	N/A	73	69	N/A	N/A
**Deal with colleagues**	Inform and supervise colleagues of the employee with cancer	73	73	N/A	35	46	N/A	N/A	N/A
**Collaborate**	Collaborate with the employee with cancer	55	N/A	N/A	75	N/A	38	40	N/A
**Create a positive work atmosphere**	Create a positive atmosphere at work, whether or not the employee with cancer is present	23	N/A	43	15	38	35	N/A	42
**Offer reintegration programmes**	Offer the employee with cancer external reintegration programmes (e.g. third-party support services or fitness programmes)	N/A	N/A	57	N/A	N/A	N/A	52	46
**Balance interests**	Try to cope as well as possible with the different interests at stake (e.g. those of the company, the employee with cancer and their colleagues)	N/A	36	33	45	N/A	N/A	N/A	N/A
**Provide time for reorientation and retraining**	Provide the employee with cancer with time for reorientation and retraining	N/A	N/A	N/A	30	N/A	N/A	24	38
**Seek balance between privacy and support**	Seek the right balance between respecting the privacy of the employee with cancer and offering them support	59	23	N/A	N/A	N/A	N/A	N/A	N/A
**Support financially**	Support the employee with cancer financially (e.g. continue to pay them during sick leave or help them with benefits applications)	N/A	N/A	N/A	N/A	N/A	46	N/A	N/A
**Comply with legislation**	Comply strictly with the obligations imposed by the law	N/A	N/A	N/A	45	N/A	N/A	N/A	N/A
**Search for external support for yourself (employer)**	Seek out external support for yourself as the employer of an employee with cancer (e.g. from the occupational physician, other employers or a psychologist). Note that this external support does not target the employee	N/A	N/A	N/A	N/A	N/A	N/A	N/A	N/A
**Possess or seek knowledge of cancer**	Possess or seek out general knowledge of cancer, its treatment and its possible consequences for work	N/A	N/A	N/A	N/A	N/A	N/A	N/A	N/A
**Additional action: support relationship with direct supervisor**	Support a good relationship between the employee with cancer and their direct supervisor	50	N/A	N/A	45	N/A	N/A	N/A	N/A

N/*A* not applicable. These employer actions were not included in the second Delphi round, either because they were selected less often during the first round or because they were not added by the experts for this specific RTW phase

Bold: the expert panel reached consensus (≥ 80%) on the importance of this employer action during this specific RTW phase

### Selection of Employer Actions

#### Employers

During Delphi round 1, the employers suggested a total of 14 additional employer actions across the four RTW phases. One additional action for RTW phases 1 and 4 met the criteria for inclusion in Delphi round 2: ‘support relationship with direct supervisor’ (see “Appendix”). The other actions were either not considered as an employer action (n = 1: ‘... the employee himself owns his work and its absence and is deemed to act from that perspective’) or already covered by another employer action (n = 11, e.g., ‘keep in touch with the employee when he or she is absent for operations and treatments’, which was covered by the employer action: ‘communicate’). These actions were therefore not included in Delphi round 2.

Consensus was reached on the importance of nine different employer actions, divided over all four RTW phases (and with some included in more than one phase): three in phase 1, *disclosure* (‘practical support’, ‘communicate’ and ‘emotional support’); four in phase 2, *treatment* (‘communicate’, ‘emotional support’, ‘allow sufficient sick leave’ and ‘plan return to work’); five in phase 3, *RTW planning* (‘assess work ability’, ‘communicate’, ‘adjust expectations’, ‘plan return to work’ and ‘show appreciation’); and five in phase 4, *actual RTW* (‘practical support’, ‘treat normally’, ‘assess work ability’, ‘show appreciation’ and ‘plan return to work’). See also Table 3.

#### Employees with Cancer

Eleven additional employer actions were suggested during Delphi round 1. Six of these were not considered as an employer action (e.g., ‘good advice and coaching from the occupational physician’) and five were already covered by another employer action (e.g., ‘ongoing communication about how the employee is doing personally and at work’, which was covered by the employer action ‘communicate’).

The employees with cancer reached consensus on the importance of eight different employer actions across the four RTW phases: four in phase 1, *disclosure* (‘allow sufficient sick leave’, ‘practical support’, ‘assess work ability’ and ‘emotional support’); two in phase 2, *treatment* (‘allow sufficient sick leave’ and ‘handle unpredictability’); five in phase 3, *RTW planning* (‘practical support’, ‘assess work ability’, ‘show appreciation’, ‘plan return to work’ and ‘adjust expectations’); and three in phase 4, *actual RTW* (‘practical support’, ‘assess work ability’ and ‘show appreciation’). See also Table 3.

## Discussion

The aim of this study was to reach consensus on which employer actions are considered most important for the RTW of employees with cancer. In this two-round Delphi study, employers reached consensus on the importance of nine employer actions: ‘emotional support’, ‘communicate’, ‘practical support’, ‘allow sufficient sick leave’, ‘plan return to work’, ‘adjust expectations’, ‘assess work ability’, ‘show appreciation’ and ‘treat normally’. Employees with cancer reached consensus on the importance of eight employer actions: ‘emotional support’, ‘allow sufficient sick leave’, ‘practical support’, ‘assess work ability’, ‘handle unpredictability’, ‘plan return to work’, ‘adjust expectations’, and ‘show appreciation’.

### Comparison with the Literature

Both stakeholder groups showed great similarity regarding perceptions of important employer actions for the RTW of employees with cancer and reached consensus on the importance of seven corresponding employer actions. For example, both agreed on the importance of emotional support during the first RTW phase, employer appreciation during the later phases, making a plan for the RTW in consultation with the employee during phase 3 and practical support during phases 1 and 4. These finding are in line with a recent qualitative study with breast cancer survivors on supporting practices of their employers, in which the importance of preparing a structured RTW before the actual RTW and the importance of flexible working hours (i.e. practical support) after RTW were mentioned [20]. Similar results were also found in a qualitative study concerning supervisor actions during the RTW of employees on sick leave due to depression [26]. This study found that emotional support, giving recognition and providing assistance (i.e. practical support) were among the most implemented employer actions to facilitate the RTW [26]. Interestingly, neither employers nor employees with cancer selected ‘possess of seek knowledge of cancer’ among the most important employer actions in the current study, even though previous studies have found repeatedly that employers do need a certain amount of knowledge of cancer in order to manage the RTW of employees with cancer [18, 19, 27]. Another study on the RTW of mixed populations also found knowledge of the consequences of the employee’s sickness for their work to be among the most important supervisor competencies [28]. That knowledge on cancer was not among the most important employer actions in the current study may be due to the high level of experience of the employers taking part: experienced employers might undervalue its importance because, for them, a certain amount of cancer-related knowledge might be ‘normal’. Whether less experienced employers would select knowledge of cancer as one of the most important employer actions and if so, what specific knowledge these employers need to facilitate the RTW of employees with cancer, should therefore be subject of further research.

Some interesting differences between stakeholder perceptions were also found, especially with regard to the phase in which the employee is on sick leave as a result of their treatment (RTW phase 2). Employers selected communicating effectively and making a RTW plan as important employer actions, whereas employees agreed on the importance of an employer being able to cope with the unpredictability of the illness. This indicates that employers might be one step ahead of the employee at this stage, a finding also substantiated by previous studies. For example, employees on sick leave with cancer might still feel vulnerable, uncertain about their mental and physical ability to work and sensitive to contact with their employer [29, 30], whereas employers are already trying to manage the absence and expected return to work of the employee [31, 32]. The discrepancy between these two perspectives could put effective employee–employer collaboration at risk. Since previous studies have perceived collaboration between the two parties as a pre-requisite for a successful RTW [30, 33], mutual understanding of their respective perspectives could well be crucial to facilitate the RTW of employees with cancer. For this reason, it is recommended that both stakeholders be open: employees with cancer about their uncertainties and needs regarding RTW, and employers about their need to be updated about the employee’s situation in order to manage the absence of the employee. For this, employers might benefit from communication skills training, since such training for employees with cancer was perceived to be helpful to enhance communication in the workplace [34]. We therefore recommend to provide employers with communication skills training to improve employer–employee communication and collaboration, with the aim of enhancing RTW of employees with cancer.

Other previous studies have shown that employers feel uncertain about what actions are required to facilitate the RTW of employees with cancer [19, 35]. Although the employers included in this study did reach consensus on the importance of a number of actions, perspectives were different for the majority of actions. This is in line with a previous study which found that organisational culture and the characteristics of both the employer and the employee might influence perceptions concerning facilitating employer actions [19]. The influence of employee characteristics was also discerned in the current study, since a certain variance was found in the selection of important employer actions by the employees with cancer, especially during RTW phase 2. This variance may be the result of differences between the employees in terms of their diagnosis and treatment [36] and in how they experience their illness and being work disabled—differences which might affect their support needs from the employer [37]. It is therefore recommended that employers be aware of the full range of actions which might facilitate the RTW of employees with cancer and tailor their use to the needs and preferences of the individual employee.

### Strengths and Limitations

The strength of the current study is its inclusion of the perspectives of both employees with cancer and employers, as well as the fact that it draws a distinction between the different RTW phases. This enhances the practical utility of the results. Secondly, the level of flexibility provided by the Delphi technique and the absence of strict guidelines resulted in a study design tailored to the current status of international research and in line with the aims of the study, e.g. with a preparatory round and concerning the predefined consensus threshold [38]. Thirdly, the participating employers had ample experience guiding employees with cancer, in terms of both years (11 on average) and numbers (about 150 in total), which contributed to the external validity of the outcomes. Finally, the heterogeneity of the employees and employers taking part also contributed to the external validity of the findings.

Some limitations should also be taken into consideration. Firstly, although the number of experts per panel was in accordance with the Delphi guidelines [25], the study’s statistical power might not be adequate to be sure that the 80% consensus threshold was not reached by chance and thereby to generalise the results to a larger population. We therefore suggest increasing the recommended number of experts per panel in order to enhance the statistical substantiality of future Delphi studies. Secondly, despite the considered process used to define the employer actions, some participants noticed a certain amount of overlap between certain actions (e.g. between ‘adjust expectation’ and ‘reduce work pressure’), which may have influenced their selection and thereby the internal validity of the findings. Thirdly, the number of actions included per RTW phase in the second Delphi round ranged between 15 and 18, due to the inclusion of additional suggested actions and a shared 15th position—the cut-off point for inclusion—in the first Delphi round. Since the experts had to select a fixed number of actions, 10, including more of these in the second Delphi round increased the number of possible permutation and so reduced the chance of reaching the ≥80% consensus threshold. Finally, two limitations might have influenced the external validity of the findings. Firstly, although we strived for heterogeneity on company size, the employers included hardly worked at small companies. The underrepresentation of small sized companies, which has also been noticed for international research at large [39], may have affected the outcomes of the current study. Large sized companies may have resources not available at smaller companies [39], for example an occupational physician to ‘assess the work ability’ of an employee with cancer, which might have resulted in an underestimation of the importance of these employer actions in this study. Besides, in the Netherlands some employer actions are required by law and this may have influenced the selection and hence the external validity of the findings for countries with different legislation. For example, Dutch employers are obliged to cover the employee’s income for at least 2 years [40]. This may be why few participants in this study selected financial support as an important employer action, whereas that might be a more relevant employer action in countries with fewer employer-related obligations from the social security system.

### Recommendations for Future Research and Practice

We recommend to study international variations in perceptions of important employer actions for the RTW of employees with cancer, since these perceptions may be influenced by national and organisational policies. With regard to practice, we recommend developing interventions that facilitate employers to perform the most important employer actions, e.g. information about appropriate practical support and assistance in assessing the work ability of an employee with cancer. These interventions should meet both employer and employee needs, so as to enhance RTW support for employees with cancer. It is also recommended that future RTW interventions draw a distinction between the four RTW phases presented in the current study, since the selection of important employer actions differed between these four phases. These differences confirm the existence of the different RTW phases and imply that each phase requires a specific approach from the employer. For phase 4, a period up until 6 months after reaching a stable situation was chosen. We do not expect that participants would have selected other employer actions when we would have chosen a longer period of follow-up. However, further research should confirm this. Lastly, some employers mentioned that they missed a fifth phase: when RTW is not possible due to the employee’s health or for organisational reasons. This phase was also identified in a recent qualitative study among employers [19], but was omitted from this study because it fell outside its particular scope, namely the *return to work* of employees with cancer. However, we still recommend the study of important employer actions during this fifth phase, since knowledge of what to do at this stage lacks and employers have mentioned to experience this as stressful phase [19].

## Conclusions

The current study ordered the wide range of findings from international qualitative studies on the role of the employer during the RTW of employees with cancer into consensus on the importance of a number of concrete employer actions in different RTW phases. Employers reached consensus on the importance of nine employer actions, employees with cancer on eight. Although the two stakeholders’ perspectives on important employer actions showed great similarities, with consensus on seven corresponding employer actions, perspectives did vary when it came to important actions while the employee is on sick leave as a result of their treatment. The results can be used to develop interventions targeting the employer, with the aim of enhancing their RTW support for employees with cancer throughout the different RTW phases. These interventions should meet both employer and employee needs, and should also incorporate a certain amount of flexibility since the employee perspectives concerning important employer actions were not univocal during all the RTW phases.

## Appendix

See Table 4.

**Table 4:** Selection of employer actions during Delphi round 1

Selection of employer actions	Expert panel	Employers				Employees with cancer			
	RTW phase	1. Disclosure	2. Treatment	3. RTW planning	4. Actual RTW	1. Disclosure	2. Treatment	3. RTW planning	4. Actual RTW
	N	22	22	21	20	26	26	25	26
Employer action	Brief explanation	Percentage of the expert panel selecting this action during Delphi round 1 as one of the 10 ‘most important’ employer actions							
**Support practically**	Provide the employee with cancer with practical support (e.g. adapting tasks, workplace and working hours)	**83**	**57**	**50**	**70**	**59**	**48**	**64**	**83**
**Assess work ability**	Assess the extent to which the employee with cancer is able to work in the right manner	**74**	**65**	**73**	**90**	**59**	**52**	**82**	**70**
**Show appreciation**	Give the employee with cancer the feeling that you want them back at work	**52**	**61**	**77**	**65**	**67**	**76**	**55**	**65**
**Communicate**	Communicate effectively with the employee with cancer (in terms of tone, intensity, subjects and channels)	**65**	**65**	**59**	**60**	**52**	**71**	**55**	**52**
**Support emotionally**	Support the employee with cancer emotionally (e.g. showing interest, being involved and understanding)	**78**	**74**	**50**	**60**	**74**	**76**	**50**	**65**
**Adjust expectations**	Adjust expectations regarding the performance of the employee with cancer to their current situation	**43**	**43**	**77**	**55**	**63**	**67**	**73**	**78**
**Allow sufficient sick leave**	Allow sufficient sick leave and not putting pressure on the employee with cancer to return to work	**65**	**70**	**45**	20	**81**	**81**	**59**	**57**
**Treat normally**	Treat the employee with cancer as if they are not ill (e.g. avoid inappropriate treatment, including being too protective or concerned)	**48**	**43**	**50**	**65**	**63**	**48**	**50**	**65**
**Plan return to work**	Make a plan for the employee’s RTW in consultation with them	22	**61**	**95**	**55**	**33**	**52**	**68**	**57**
**Handle unpredictability**	Try to cope as well as possible with the unpredictability of the illness and the absence of the employee with cancer	30	**61**	**41**	**45**	**52**	**57**	**55**	**57**
**Reduce work pressure**	Reduce the pressure of work on the employee with cancer	**39**	22	**41**	**50**	**67**	**52**	**50**	**61**
**Radiate a positive attitude**	Radiate a positive attitude when guiding the employee with cancer	**48**	**48**	**55**	**35**	**48**	**43**	**45**	**43**
**Respect privacy**	Respect the privacy of the employee with cancer	**43**	**48**	23	25	**56**	**48**	23	13
**Deal with colleagues**	Inform and supervise colleagues of the employee with cancer	**61**	**74**	32	**30****	**37**	24	23	22
**Collaborate**	Collaborate with the employee with cancer	**52**	30	23	**75**	26	**33***	**41**	35
**Create a positive work atmosphere**	Create a positive atmosphere at work, whether or not the employee with cancer is present	**35**	22	**50**	**30****	**44**	**38**	32	**48**
**Offer reintegration programmes**	Offer the employee with cancer external reintegration programmes (e.g. third-party support services or fitness programmes)	9	26	**41**	25	15	19	**45**	**39**
**Balance interests**	Try to cope as well as possible with the different interests at stake (e.g. those of the company, the employee with cancer and their colleagues)	30	**39**	**41**	**45**	15	14	18	13
**Provide time for reorientation and retraining**	Provide the employee with cancer with time for reorientation and retraining	4	4	14	**30****	4	5	**36**	**39**
**Seek balance between privacy and support**	Seek the right balance between respecting the privacy of the employee with cancer and offering them support	**48**	**39**	18	20	30	24	18	17
**Support financially**	Support the employee with cancer financially (e.g. continue to pay them during sick leave or help them with benefits applications)	13	9	5	10	15	**33***	14	4
**Comply with legislation**	Comply strictly with the obligations imposed by the law	30	26	27	**35**	15	19	18	9
**Search for external support for yourself (employer)**	Seek out external support for yourself as the employer of an employee with cancer (e.g. from the occupational physician, other employers or a psychologist). Note that this external support does not target the employee	17	4	14	0	4	10	18	4
**Possess or seek knowledge of cancer**	Possess or seek out general knowledge of cancer, its treatment and its possible consequences for work	9	9	0	5	22	10	9	4
**Additional actions**		Support relationship with direct supervisor	–	–	Support relationship with direct supervisor	–	–	–	–

Bold: The expert panel selected this employer action among the 15 most important actions, during this specific RTW phase. These actions were therefore included in the second Delphi round

*/**These employer actions were selected on place 15 and 16 (*) or 15, 16 and 17 (**), but were selected by the same percentage of the expert panel and were therefore both/all included in the second Delphi round
